# Risk factors for unsuppressed viral load after intensive adherence counseling among HIV infected persons in Kampala, Uganda: a nested case–control study

**DOI:** 10.1186/s12981-023-00583-3

**Published:** 2023-12-19

**Authors:** Jonathan Izudi, Barbara Castelnuovo, Rachel King, Adithya Cattamanchi

**Affiliations:** 1 Infectious Diseases Institute, Makerere University College of Health Sciences, Kampala, Uganda; 2 University of California Global Health Institute, University of California San Francisco, San Francisco, CA USA; 3 Department of Epidemiology and Biostatistics, Institute for Global Health Sciences, University of California, San Francisco, San Francisco, CA USA; 4 Division of Pulmonary Diseases and Critical Care Medicine, University of California Irvine, Irvine, CA USA

**Keywords:** Detectable viral load, Intensive adherence counseling, Viral load suppression

## Abstract

**Background:**

Intensive adherence counseling (IAC) is the global standard of care for people living with human immunodeficiency virus (PLHIV) who have unsuppressed VL after ≥ 6 months of first-line anti-retroviral therapy (ART). We investigated whether the number of IAC sessions is associated with suppressed VL among PLHIV in Kampala, Uganda.

**Methods:**

We conducted a nested case-control study among PLHIV with unsuppressed VL after ≥ 3 IAC sessions (cases) and a 2:1 random sample of PLHIV with suppressed VL after ≥ 3 IAC sessions (controls). Unsuppressed VL was defined as VL ≥ 1000 copies/ml. We performed multivariable logistic regression to identify factors that differed significantly between cases and controls.

**Results:**

Demographic and clinical characteristics were similar among the 16 cases and 32 controls including mean age, sex, baseline CD4 count, VL before IAC, and WHO clinical stage. Only the number of IAC sessions differed significantly between cases and controls in unadjusted (p = 0.012) and adjusted (p = 0.016) analyses. Each unit increase in IAC session was associated with unsuppressed VL (Adjusted odds ratio 5.09; 95% CI 1.35–19.10).

**Conclusions:**

VL remained unsuppressed despite increasing IAC frequency. The fidelity to standardized IAC protocol besides drug resistance testing among PLHIV with unsuppressed VL before IAC commencement should be examined.

## Background

With optimal ART adherence, nearly all people living with human immunodeficiency virus (PLHIV) achieve viral load (VL) suppression within 6 months of anti-retroviral therapy (ART) initiation. For those with unsuppressed VL, an ART adherence intervention is needed [1]. The World Health Organization (WHO) recommends intensive adherence counseling (IAC) for PLHIV with unsuppressed VL before diagnosing treatment failure and switching to a second-line ART. IAC consists of three targeted and structured counseling and support sessions, spaced 1 month apart, provided by a multidisciplinary team to overcome barriers to ART adherence [2, 3].

Past studies in Uganda [2, 4, 5] show that a significant proportion of PLHIV with an unsuppressed VL never achieve VL suppression even after ≥ 3 IAC sessions but this problem has not been extensively studied. Our study investigated whether the number of IAC sessions is associated with suppressed VL among adolescents and adults living with HIV on first-line ART who had unsuppressed VL after 6 or more months on ART in Kampala, Uganda.

## Methods

This was a sub-analysis of data from the EFFINAC study [6] that retrieved medical records across six public Kampala Capital City Authority (KCCA) health facilities described previously [7, 8]. The EFFINAC study evaluated the impact of IAC on VL suppression and mortality among PLHIV on first-line ART, with the intervention group as those who had received ≥ 3 consecutive IAC sessions provided 1 month apart (n = 114) and comparison as those who received psychosocial support (n = 3085). The study sites provide standardized HIV/ART care following the national treatment guidelines. The first VL testing is done after 6 months of ART initiation and subsequent tests are done annually if one is virally suppressed. If one is not virally suppressed, IAC is provided according to guidelines. The parent study received ethical approvals from the Infectious Diseases Institute Research Ethics Committee (#IDI-REC-2022-18) and the Uganda National Council for Science and Technology (#HS25553ES), and administrative clearance from the Directorate of Public Health and Environment, KCCA (#DPHE/KCCA/1301). The study considered participants initiated on first-line ART between November 1, 2020, and November 30, 2021, with the data retrieval period as November 1, 2022, to January 5, 2023. For this nested case-control study, the IDI-REC and UNCST provided a waiver of informed consent since the study was embedded within the parent study [6]. PLHIV aged ≥ 15 years with repeat unsuppressed VL (VL ≥ 1000 copies/ml) after ≥ 3 IAC sessions were considered as cases (n = 16) and a 2:1 random sample of those with repeat suppressed VL (VL < 1000 copies/ml) were selected as controls (n = 32).

We excluded PLHIV that transferred to other health facilities and those that died before a repeat VL testing. We summarized numerical data using mean and standard deviation (when normally distributed) and categorical data using frequencies and percentages. Bivariate analysis used Fisher’s exact test to assess differences in proportions between cases and controls. Mean differences in numerical data between cases and controls were assessed using Student’s t-test for normally distributed data, otherwise, the Wilcoxon-rank sum test was used. Socially and clinically relevant variables from the literature and those with p < 0.1 at the bivariate analysis were included in the multivariable logistic regression analysis to determine whether the number of IAC sessions is associated with suppressed VL. We reported odds ratio (aOR) and 95% confidence interval (CI).

## Results

Of 114 PLHIV that received ≥ 3 IAC sessions, 67 had repeat VL testing of whom 16 had unsuppressed VL (cases) while 51 had suppressed VL. From the latter category, we randomly sampled 32 participants as controls, yielding a 1:2 case-to-control ratio. Table 1 summarises the participants’ characteristics. On average, cases and controls had comparable mean ages: 26.2 ± 15.7 versus 27.8 ± 9.8 respectively, p = 0.67. We found a borderline difference between cases and controls regarding baseline ART regimen (p = 0.058) but a significant difference concerning the frequency of IAC sessions (P = 0.009). Other variables showed no difference between cases and controls. Each unit increase in IAC frequency was significantly associated with being a case in unadjusted (OR, 4.53; 95% CI 1.39–14.74) and adjusted (aOR, 5.09; 95% CI 1.35–19.10) analyses (Table 2).

**Table 1:** General characteristics of cases and controls

Variables	Level	Cases (n = 16)	Controls (n = 32)	P-value
Study site	Kawaala HC	2 (12.5)	1 (3.1)	0.122
	Kisenyi HC	9 (56.2)	13 (40.6)	
	Kisugu HC	0 (0.0)	1 (3.1)	
	Kiswa HC	1 (6.2)	3 (9.4)	
	Kitebi HC	0 (0.0)	10 (31.2)	
	Komamboga HC	4 (25.0)	4 (12.5)	
Level of health facility	HC III	7 (43.8)	19 (59.4)	0.473
	HC IV	9 (56.2)	13 (40.6)	
Age group (years)	15–24	7 (43.8)	11 (34.4)	0.212
	25–34	4 (25.0)	16 (50.0)	
	35 and more	5 (31.2)	5 (15.6)	
	Mean (SD)	26.2 (15.7)	27.8 (9.8)	0.674
Sex	Female	11 (68.8)	24 (75.0)	0.909
	Male	5 (31.2)	8 (25.0)	
Point of entry into HIV care	Others	0 (0.0)	3 (9.4)	0.471
	Outpatient department	10 (62.5)	15 (46.9)	
	PMTCT clinic	6 (37.5)	13 (40.6)	
	TB clinic	0 (0.0)	1 (3.1)	
Mid-upper arm circumference	Green (well-nourished)	15 (93.8)	32 (100.0)	0.721
	Yellow (moderately malnourished)	1 (6.2)	0 (0.0)	
Baseline CD4 (cells/ml)	500 and more	13 (81.2)	27 (84.4)	1.000
	Less than 500	3 (18.8)	5 (15.6)	
VL before IAC (copies/ml)	Less or equal to 10,000	8 (50.0)	11 (34.4)	0.465
	More than 10,000	8 (50.0)	21 (65.6)	
ART regimen	ABC-3TC-EFV	3 (18.8)	2 (6.2)	0.058
	ABC/3TC/DTG	1 (6.2)	0 (0.0)	
	Other first-line regimens	2 (12.5)	0 (0.0)	
	TDF-3TC-EFV	0 (0.0)	1 (3.1)	
	TDF/3TC/DTG	10 (62.5)	29 (90.6)	
Baseline WHO clinical stages	I and II	15 (93.8)	31 (96.9)	1.000
	III and IV	1 (6.2)	1 (3.1)	
Frequency of IAC	3	6 (37.5)	26 (81.2)	0.009
	4	9 (56.2)	5 (15.6)	
	5	1 (6.2)	1 (3.1)	

*3TC* lamivudine, *ABC* abacavir, *DTG* dolutegravir, *EFV* efavirenze, *HC* Health Center, *LPV/r* lopinavir/ritonavir, *PMTCT* prevention of mother to child transmission of HIV, *TB* tuberculosis, *TDF* tenofovir

**Table 2:** Risk factors for unsuppressed VL among PLHIV

Variables	Level	Logistic regression analysis	
		Unadjusted	Adjusted
		OR (95% CI)	aOR (95% CI)
Age group (years)	15–24	1	1
	25–34	0.39 (0.09, 1.67)	0.52 (0.10, 2.73)
	35 and more	1.57 (0.33, 7.48)	2.32 (0.35, 15.18)
Sex	Female	1	1
	Male	1.36 (0.36, 5.13)	1.41 (0.28, 7.09)
Baseline CD4 (cells/ml)	500 and more	1	1
	Less than 500	1.25 (0.26, 6.03)	1.65 (0.27, 10.10)
VL before IAC (copies/ml)	Less or equal to 10,000	1	1
	More than 10,000	0.52 (0.15, 1.78)	0.51 (0.12, 2.23
Baseline WHO clinical stage	Stage I and II	1	1
	Stage III and IV	2.07 (0.12, 35.36)	1.19 (0.04, 38.82)
Increasing the frequency of IAC sessions	1-unit increase	4.53* (1.39, 14.74)	5.09* (1.35, 19.10)

Odds ratio are exponentiated coefficients at a 5% level of statistical significance; 95% confidence intervals in brackets

Bold values denote a statistically significant finding

*p < 0.05

## Discussion

VL remained unsuppressed despite increasing IAC frequency among PLHIV who had received ≥ 3 IAC sessions. Our findings suggest IAC is ineffective in halting and reversing unsuppressed VL consistent with our finding of a lack of impact of IAC on VL suppression [6]. A Swaziland study showed that VL suppression is not associated with the frequency of IAC sessions as no difference in VL suppression was observed between those who received 1–3 IAC sessions and those with zero IAC [9]. Inadequacies in ART adherence during IAC have been reported to increase the odds of unsuppressed VL [10] and might explain the finding.

However, whether cases had suboptimal ART adherence than the controls despite increasing IAC frequency remains unknown as all participants reported good ART adherence. HIV drug resistance could be another factor as recent evidence shows an increasing trend in drug resistance in pre-treated populations [11]. In South Africa, a substantial proportion of PLHIV with unsuppressed VL after IAC (41/48) had drug resistance [12]. A previous study involving 113 PLHIV on long-term ART who completed IAC in eastern Uganda found more than nine in 10 had unsuppressed VL and drug resistance testing on 105 of the participants revealed 103 (98%) had at least one mutation [4]. HIV drug resistance testing was not routinely performed at the time of our study. However, our data support that drug resistance testing should be done for PLHIV with unsuppressed VL before IAC initiation as IAC will not achieve VL suppression in the presence of drug resistance.

Our study has strengths and limitations to consider. We analyzed data on cases and controls drawn from the same cohort, making the two groups almost similar on several measured factors. Limitations include a small sample size so the evidence should be considered preliminary. We analyzed secondary data so data on HIV drug resistance and IAC implementation fidelity that might explain unsuppressed VL were not available. Future, prospective studies should account for these factors and further explore socio-behavioral factors (e.g., stigma, discrimination, social support) that might contribute to sub-optimal ART adherence and potential worsening of adherence as a result of IAC.

## Conclusions and recommendations

An increase in the number of IAC sessions did not achieve VL suppression. We recommend a need to examine the fidelity of IAC implementation and to perform drug resistance testing among PLHIV with unsuppressed VL before IAC initiation and a switch to second or third-line ART.

## Data Availability

The datasets used and/or analysed during the current study are available from the corresponding author on reasonable request.

## Abbreviations

ART: Anti-retroviral therapy
HIV: Human immunodeficiency virus
IAC: Intensive adherence counseling
PLHIV: People living with HIV
VL: Viral load
